# Objectively measured environmental features and the risk of dementia: a systematic review and meta‐analysis

**DOI:** 10.1002/alz.091425

**Published:** 2025-01-09

**Authors:** Suhang Song, Zimu Jia, Nicholas Gary Lamont Myers, Jin Sun

**Affiliations:** ^1^ University of Georgia, Athens, GA USA; ^2^ The College of Wooster, Wooster, OH USA

## Abstract

**Background:**

Existing evidence supports that living in a friendly environment is associated with lower risk of dementia, although the measurements of environmental variables are mixed. However, the type of environmental features reducing dementia risk has not been comprehensively identified and such associations have not been quantified. This systematic review and meta‐analysis aims to summarize the objectively measured environmental features associated with dementia and examine the pooled association between those features and dementia risk in older adults.

**Method:**

We systematically searched PubMed, Web of Science, and EBSCO (Abstracts in Social Gerontology, AgeLine, APA PsycINFO, and CINAHL) databases on December 21, 2023, to identify the empirical studies assessing the role of objectively measured environmental features in the risk of dementia. The search yielded a total of 890 citations. According to the inclusion and exclusion criteria, ten studies were included in the systematic review and meta‐analysis. Among them, four articles included more than one environmental features, resulting in a total of 15 associations between environmental variables and dementia risk.

**Result:**

This study summaries five environmental features that have been reported to be associated with lower dementia risk, including longer distance between residence and major roads (Hazard Ratio [HR] = 0.94, 95% Confidence Interval[CI] = 0.88‐1.00), larger green space (HR = 0.94, 95%CI = 0.88‐1.00), higher level of sidewalk installation (HR = 0.71, 95%CI = 0.52‐0.90), and less exposure to air pollution (i.e., NO2 [HR = 1.05, 95%CI = 0.96‐1.15], PM2.5 [HR = 1.03, 95%CI = 1.02‐1.05], and PM10 [HR = 1.13, 95%CI = 1.05‐1.21]). Pooled analysis showed that living in a friendly environment may reduce 7% of the risk of dementia (HR = 0.93, 95%CI = 0.90‐0.97) and exposure to air pollution may increase 5% of the dementia risk (HR = 1.05, 95%CI = 1.02‐1.08). There was heterogeneity in results across studies.

**Conclusion:**

Even though a small number of studies included in this systematic review and meta‐analysis have examined the association between objectively measured environmental features and dementia risk, all existing investigations point to statistically significant associations in older adults. Whether those associations vary in different population subgroups and the mechanisms underlying those associations warrant further investigation.

